# Combined quantitative measures of ER, PR, HER2, and KI67 provide more prognostic information than categorical combinations in luminal breast cancer

**DOI:** 10.1038/s41379-019-0270-4

**Published:** 2019-04-11

**Authors:** Mustapha Abubakar, Jonine Figueroa, H. Raza Ali, Fiona Blows, Jolanta Lissowska, Carlos Caldas, Douglas F. Easton, Mark E. Sherman, Montserrat Garcia-Closas, Mitch Dowsett, Paul D. Pharoah

**Affiliations:** 1 Division of Cancer Epidemiology and Genetics, National Cancer Institute, National Institutes of Health, Rockville, MD USA; 2 Division of Genetics and Epidemiology, The Institute of Cancer Research, London, UK; 3 Usher Institute of Population Health Sciences and Informatics, The University of Edinburgh, Scotland, UK; 4 Cancer Research UK (CRUK) Cambridge Institute, University of Cambridge, Cambridge, UK; 5 Center for Cancer Genetic Epidemiology, Department of Oncology, University of Cambridge, Cambridge, UK; 6 Department of Cancer Epidemiology and Prevention, M. Sklodowska-Curie Memorial Cancer Center and Institute of Oncology, Warsaw, Poland; 7 Department of Oncology, University of Cambridge, Cambridge, UK; 8 Cambridge Experimental Cancer Medicine Centre and NIHR Cambridge Research Centre, Cambridge, UK; 9 Centre for Cancer Genetic Epidemiology, Department of Public Health and Primary Care, University of Cambridge, Cambridge, UK; 10 Division of Epidemiology, Department of Health Sciences Research, Mayo Clinic, Jacksonville, FL USA; 11 Breast Cancer Now Toby Robins Research Centre, Division of Breast Cancer Research, The Institute of Cancer Research, London, UK; 12 Academic Department of Biochemistry, Royal Marsden Hospital, Fulham Road, London, UK

**Keywords:** Breast cancer, Prognostic markers, Prognostic markers

## Abstract

Although most women with luminal breast cancer do well on endocrine therapy alone, some will develop fatal recurrence thereby necessitating the need to prospectively determine those for whom additional cytotoxic therapy will be beneficial. Categorical combinations of immunohistochemical measures of ER, PR, HER2, and KI67 are traditionally used to classify patients into luminal A-like and B-like subtypes for chemotherapeutic reasons, but this may lead to the loss of prognostically relevant information. Here, we compared the prognostic value of quantitative measures of these markers, combined in the IHC4-score, to categorical combinations in subtypes. Using image analysis-based scores for all four markers, we computed the IHC4-score for 2498 patients with luminal breast cancer from two European study populations. We defined subtypes (A-like (ER + and PR + : and HER2- and low KI67) and B-like (ER + and/or PR + : and HER2 + or high KI67)) by combining binary categories of these markers. Hazard ratios and 95% confidence intervals for associations with 10-year breast cancer-specific survival were estimated in Cox proportional-hazard models. We accounted for clinical prognostic factors, including grade, tumor size, lymph-nodal involvement, and age, by using the PREDICT-score. Overall, Subtypes [hazard ratio (95% confidence interval) B-like vs. A-like = 1.64 (1.25–2.14); *P*-value < 0.001] and IHC4-score [hazard ratio (95% confidence interval)/1 standard deviation = 1.32 (1.20–1.44); *P*-value < 0.001] were prognostic in univariable models. However, IHC4-score [hazard ratio (95% confidence interval)/1 standard deviation = 1.24 (1.11–1.37); *P*-value < 0.001; likelihood ratio chi-square (LR*χ*^2^) = 12.5] provided more prognostic information than Subtype [hazard ratio (95% confidence interval) B-like vs. A-like = 1.38 (1.02–1.88); *P*-value = 0.04; LR*χ*^2 ^= 4.3] in multivariable models. Further, higher values of the IHC4-score were associated with worse prognosis, regardless of subtype (*P*-heterogeneity = 0.97). These findings enhance the value of the IHC4-score as an adjunct to clinical prognostication tools for aiding chemotherapy decision-making in luminal breast cancer patients, irrespective of subtype.

## Introduction

Breast cancer is the most common malignancy and the leading cause of cancer-related mortality among women worldwide [1]. With the advent and increasing uptake of screening programs, the incidence of early stage, hormone receptor positive (HR + )/luminal-like [estrogen receptor positive (ER + ) and/or progesterone receptor positive (PR + )] breast cancer has continued to rise [2, 3]. Accounting for almost 70% of all cases, luminal-like tumors comprise the majority of breast cancers in Western populations [4]. These tumors are notably heterogeneous, encompassing subtypes with distinct molecular profiles and clinical outcomes [5–8]. Based on gene expression profiling, two main subtypes of luminal-like tumors have been identified. Denoted as luminal A and B, these subtypes are differentiated by their relative expressions of hormone and proliferation-related genes and, in a subset of luminal B tumors, by the amplification of the human epidermal growth factor receptor 2 (HER2/neu) gene [5, 9].

Although most women with luminal-like disease do well on endocrine therapy alone, some of them develop fatal recurrence thereby necessitating the need for additional cytotoxic therapy. Owing to the debilitating side-effects associated with chemotherapy, the need to prospectively distinguish women for whom its addition will be beneficial from those for whom this may not be needed remains a challenge in translational breast cancer research [10]. Several prognostic tools have been developed to address this, including those that rely on standard clinical prognostic factors [11–13] and others, such as the IHC4-score, that are based on immunohistochemical measures on ER, PR, HER2, and KI67 [13]. In addition, some international guidelines have endorsed the use of immunohistochemistry-based (i.e., luminal A-like and B-like) subtypes together with multiparameter molecular tests [14–17] to aid chemotherapy decision-making [18–20]. However, because standard immunohistochemistry-based luminal A-like/B-like subtype definition is based on dichotomous categories of the individual immunohistochemical markers, this may lead to the loss of prognostically relevant information. Nonetheless, it remains unclear whether quantitative measures of ER, PR, HER2, and KI67 provide more prognostic information than categorial combinations in breast cancer subtypes.

Multiparameter molecular testing is one method of quantification of hormone and proliferation-related genes that has been shown to be prognostic in breast cancer [14–17], but this is expensive, and it remains unclear whether it improves the prognostication of clinical tools such as PREDICT. PREDICT [11, 12] is a popular, breast cancer prognostication, and treatment benefit tool that remains the only breast cancer prognostication tool to be endorsed by the American Joint Committee on Cancer to date. Like traditional clinical prognostic factors in PREDICT, immunohistochemical markers are cheap to perform, widely available, and are typically assessed as part of the routine workup for most breast cancer patients. It has previously been shown that combined visual assessments of ER, PR, HER2, and KI67 in the IHC4-score provided additional prognostic information to standard clinicopathological factors and contained comparative prognostic information to the 21-gene (Oncotype DX) panel test [13].

Owing to the limitations of visual scoring, particularly for KI67 [21, 22], automated methods have been suggested as potential alternatives. We have previously demonstrated the independent prognostic value of automated scores for ER, PR, HER2, and KI67 separately, but it remains unclear whether combining these in the IHC4-score algorithm will provide additional prognostic information to immunohistochemistry-based subtypes or other clinical prognostic factors. Further, although patients with luminal A-like breast cancer generally have better clinical outcomes than those with luminal B-like disease [5, 9, 16], it is unknown if the dynamic range of the IHC4-score could be leveraged to further stratify these patients into clinically relevant subgroups for treatment decision-making.

Our primary aim in this study was, therefore, to investigate the comparative prognostic performance of image analysis-based, quantitative, measures of ER, PR, HER2, and KI67, combined in the IHC4-score, vs. categorical combinations of these markers in luminal (A-like/B-like) breast cancer subtype. As a secondary aim, we evaluated the prognostic significance of the image analysis-based IHC4-score in relation to clinical prognostic factors, combined in the clinical treatment score (*C*-score) and PREDICT-score.

## Materials and Methods

### Study population

**Fig. 1:**
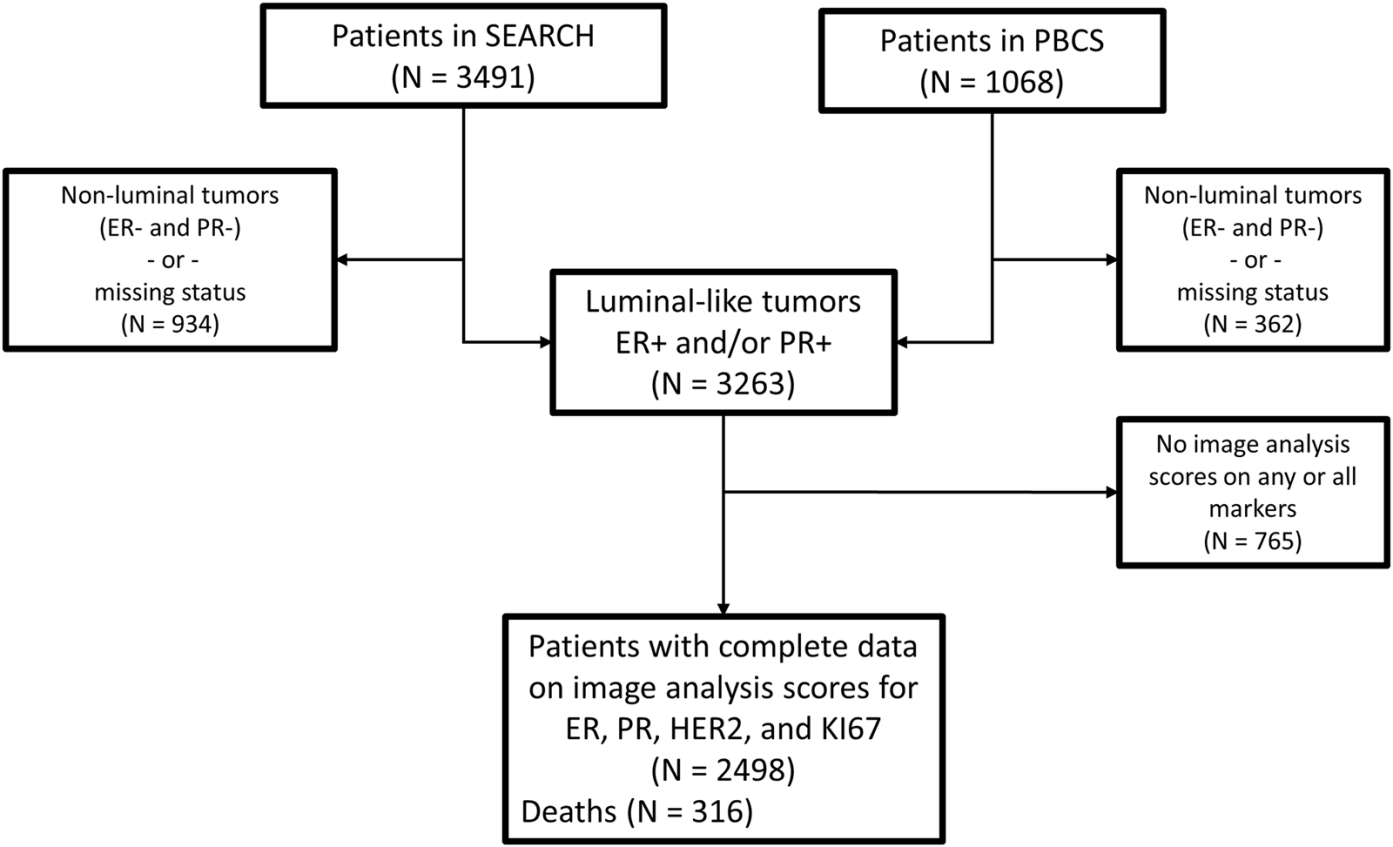
Consort diagram indicating the total number of patients included in this analysis. The patients were women with clinically determined invasive luminal-like breast cancer from two study populations in Europe (the Polish Breast Cancer Study (PBCS) in Poland and the Study of Epidemiology and Risk Factors in Cancer Heredity (SEARCH) in the United Kingdom) with complete information on image analysis-based scores of ER, PR, HER2, and KI67, as well as other relevant clinicopathological and follow-up data

The current analysis included 2498 patients with luminal-like invasive breast cancer from two study populations from Poland (*N* = 558) and the United Kingdom (*N* = 1940). The analysis comprised of women with luminal-like, i.e., ER + and/or PR + tumors and for whom we also had complete data on image analysis-based scores for ER, PR, HER2, and KI67 (Fig. 1). These scores were obtained by digital image analysis of tissue microarrays and analyzed as part of other projects [23–25] within the Breast Cancer Association Consortium. Details of both study populations have been previously described [26, 27], but in brief: The Polish Breast Cancer Study is a population-based study in Poland that enrolled women 20–74 years with histologically or cytologically confirmed breast cancer at five participating hospitals in Warsaw and Lodz over a three-year period between 2000 and 2003 [27]. The Study of Epidemiology and Risk Factors in Cancer Heredity (SEARCH) is a population-based study that began in the UK in 1996 [26]. Patients were ascertained through the Eastern Cancer Registration and Information Center and included women < 55 years of age diagnosed with invasive breast cancer between 1991 and mid-1996 who were alive at the start of the study and those < 70 years who were diagnosed from mid-1996 onwards.

Data on relevant clinicopathological characteristics, including ER, PR, HER2, histologic grade, tumor size, nodal involvement, endocrine therapy, and systemic therapy were obtained from clinical records. Patients were followed up from recruitment for the development of outcomes of interest, i.e., breast cancer-specific deaths. Among the patients included in this analysis, a total of 316 breast cancer-specific deaths (*N* = 255 and 61, for the SEARCH and Polish breast cancer studies, respectively) occurred over a median follow-up period of 7.05 years (8.01 and 5.0 years for the SEARCH and Polish study populations, respectively). In both studies, deaths were ascertained through linkage to registries, as well as by curating clinical records. Ethical approvals were obtained from local ethics committees and all participants provided written informed consent.

### Immunostaining and scoring of tissue microarrays for ER, PR, HER2, and KI67

Staining for all four markers was performed in the respective study groups by using standard laboratory techniques (Supplementary Table 1). Tissue microarray sections for ER and PR were stained by using mouse monoclonal antibodies 6F11/2 (Novocastra) and PR636 (Dako) clones, respectively, while tissue microarrays for HER2 and KI67 were stained using Herceptest kit K5207 (Dako) and MIB-1 (Dako), respectively. Dichotomous categories (positive and negative) of ER, PR, and HER2 were obtained from clinical records. ER and PR were scored using the Allred scoring method and values > 2 ( > 10% positive cells) were considered positive. For HER2, 3 + on immunohistochemistry or HER2 amplification on fluorescent in situ hybridization were HER2 + . Quantitative measures on these markers were generated by using digital pathology image analysis performed in two institutions in the UK: the Cancer Research Institute in Cambridge and the Institute of Cancer Research (ICR) in London. ER, PR, and HER2 were scored in Cambridge while KI67 was scored at the ICR. Both institutions used the Ariol machine (Leica Biosystems, Newcastle UK) for scoring. Ariol has functionality for the automatic separation of malignant and non-malignant cells based on their shape and size characteristics and, by using color deconvolution, it can detect (3–3'-diaminobenzidine) positive and negative (hematoxylin) staining malignant epithelial cells. Details of the optimized Ariol algorithms and protocols that were used for the scoring of each of these four markers have been previously described [23, 24]. In brief, for ER, PR, and KI67 nuclear staining, the Ariol system was tuned to distinguish between malignant and non-malignant cells and to count positively and negatively staining malignant cells. Based on the number of positive and number of negative tumor nuclei presented by the machine, the percentage of cells stained (0–100%) was calculated as the ratio of positive nuclei to the sum of positive and negative nuclei per tissue core. For HER2, the US Food and Drug Administration-approved Herceptest score [28] (0, 1 + , 2 + , 3 + ), generated to American Society of Clinical Oncology/College of American Pathologists guidelines was calculated by the system. As previously reported [23, 24], we observed good agreement with standardized pathologists scores for ER (observed agreement = 90%; kappa = 0.76), PR (observed agreement = 84%; kappa = 0.66), HER2 (observed agreement = 90%, kappa = 0.69), and KI67 (observed agreement = 87%; kappa = 0.64).

### Subtype definition based on binary categories of ER, PR, HER2, and KI67

Subtypes were defined according to the published St Gallen criteria [18] as follows: Luminal A-like: tumors that homogeneously expressed ER and PR (i.e., ER + and PR + ) in addition to being HER2– and low proliferating (image analysis-based KI67 ≤ 12%). We have previously reported a cutoff point of 12% for image analysis-based KI67, which corresponded to a visual score of 25%, to provide the best discrimination in terms of survival in this population [25], hence its adoption here. The luminal B-like subtype comprised tumors that were: (a) ER + and/or PR + and high proliferating (image analysis-based KI67 > 12%); (b) ER + and/or PR + and HER2 + .

### Quantitative IHC4-score generation

The average score for ER, PR, HER2, and KI67 across the total number of cores per patient was taken as the patient’s score on each marker. IHC4-scores were generated using the published algorithm [13]:

$${{{{\mathrm{IHC4-score}}}} = \, 94.7 \times \left\{ \left( { - 0.100\;{{\mathrm{{ER}}}}10} \right) + \left( { - 0.079\;{{\mathrm{{PR}}}}10} \right) \right.} \\ \hskip 50pt {\left. + \, \left( {0.586\;{{\mathrm{{HER}}}}2} \right) + \left[ {0.240\;{\mathrm{ln}}\left( {1 + 10 \times {{\mathrm{{Ki}}}}{{67}}} \right)} \right] \right\}}$$

The ER10 variable was calculated by dividing the ER% score for each patient by a factor of 10 to generate a range of values 0–10. In the original algorithm, the ER10 variable was generated by dividing the *H*-score (30–300) by a factor of 30 to give values ranging from 1–10. All other components of the IHC4-score were the same as in the published algorithm [13].

### Clinical prognostic factors

We accounted for standard clinical prognostic factors, including age at diagnosis, tumor size, histologic grade, and number of lymph nodes involved using two methods. The first was based on the *C*-score reported by Cuzick et al. [13]:

$$	{{{C-{\mathrm{score}}}} = 100 \times \{ \left( {0.417N_{1 - 3}} \right) + \left( {1.566N_{4 + }} \right)} \\ 	\hskip 10pt {+ \, [0.930 \times \left( {0.497T_{1 - 2}} \right) + \left( {0.882T_{2 - 3}} \right) + (1.838T_{ > 3})} \\ 	\hskip 10pt {+ \, \left( {0.559{\mathrm{Gr}}_{\mathrm{2}}} \right) + \left( {0.970{\mathrm{Gr}}_3} \right) + \left( {0.130{\mathrm{Age}}_{ \ge 65}} \right) + \left( {0.149{\mathrm{AI}}} \right)]\}}$$

Where *N* is the number of nodes (0, 1–3, 4 + ), *T* is tumor size ( ≤ 1 cm, > 1 to ≤ 2 cm, > 2 to ≤ 3 cm, > 3 cm), Gr is grade (1–3) and Age being the patients age at diagnosis ( < 65, ≥ 65 years). Data on specific treatment regimen received by each patient were not available; as such, the aromatase inhibitor (AI) vs. tamoxifen component was not computed.

The second was based on the parameters used for the PREDICT prognostication model:

$$	{\mathrm{PREDICT-score}} ({\mathrm{ER}} + ) = (34.53 ({\mathrm{Age}}/10)^{ - 2} - 0.0287)\\ 	 + ( - 34.20 ( {\mathrm{Age}}/10 )^{ - 2} \times {\mathrm{log}} ( {\mathrm{Age}}/10 ) - 0.0510 ) \\ 	 + ( 0.7531 \times {\mathrm{log}} ( {\mathrm{Size}}/100) + 1.5452 ) \\ 	 + (0.7069 \times {\mathrm{log}} (( {\mathrm{Nodes}} + 1)/10 ) + 1.3876 ) \\ 	 + (0.7467 ({\mathrm{Grade}}) ) + ( - 0.2763 ( {\mathrm{Screen}}-{\mathrm{detected}} ))$$

We did not have information on mode of detection, therefore, could not compute the screen-detected vs. interval (or non-screen-detected) component of the PREDICT-score. Notably, organized mammography screening was not available in Poland during the study. All other components were as in the original equation.

### Statistical analysis

Participant’s ages were categorized as < 35, 35–50, 50–65, and > 65 years. Chi-square, for categorical variables, and non-parametric Kruskal–Wallis tests, for continuous variables, were used to assess the frequencies of tumor clinicopathological characteristics (including age at diagnosis, histologic grade, stage, morphology, size, lymph-nodal status, and treatment), overall and by study population. Histograms and box plots were used to assess the distribution of the IHC4-score, overall and by study population. In univariable Cox proportional hazard regression models, we assessed the associations between subtype (B-like vs. A-like) and continuous measures of the IHC4-score, *C*-score, and PREDICT-score with 10-year breast cancer-specific survival. Additionally, for subtypes (luminal A-like and B-like) and quartiles (Q1–Q4) of the IHC4-score, we examined 10-year breast cancer-specific survival in Kaplan–Meier survival curves. For the IHC4-score this analysis was further stratified by nodal status (i.e., node-negative and node-positive). In multivariable Cox proportional models, we adjusted for study population (in combined analysis), treatment, and other standard clinical factors, including age at diagnosis, tumor size, histologic grade, and lymph-nodal involvement. These features were combined by means of the *C*-score and PREDICT-score. The relative contributions of the *C*-score and PREDICT-score to a prognostic model were determined by assessing the change in likelihood ratio chi-square (∆LR*χ*^2^) when either of these was removed from the full model. We used loglikelihood and LR*χ*^2^ values to compare model fit between prognostic scores. All analyses were performed overall and following stratification by study population. In subtype-specific analysis, we examined the prognostic value of the IHC4-score within each of the luminal-like breast cancer subtypes. To determine whether automated IHC4-score can be used to further stratify luminal-like breast cancer patients into prognostically relevant subgroups, we dichotomized the IHC4-score at the mean + 1 standard deviation threshold and examined associations with 10-year breast cancer-specific survival in Kaplan–Meier curves and in multivariable Cox proportional hazard models. Violations of the proportionality assumption of the Cox model were assessed by modeling the predictors as time-varying covariates. As part of sensitivity analysis, we redefined luminal-like breast cancer subtype by using a cut-off point of ≥ 1% on ER and PR [20] and examined the prognostic value of the IHC4-score in the resulting luminal A-like and B-like subtypes. Owing to the low prevalence of chemotherapy in this population (~7%), we could not perform analyses stratified by chemotherapy; instead, the few women who received chemotherapy (*N* = 178) were excluded from the survival analysis. All tests were two-sided, and analyses were conducted using Stata statistical software version 14.1 (StataCorp, Lakeway Drive, TX, USA).

## Results

### Description of study population

As shown in Table 1, the majority (86%) of the patients were between the ages of 35 and 65 years at diagnosis, with women from the SEARCH study being younger than those from the Polish study on average (*P*-value < 0.001). Overall, and in both study populations, most of the tumors (82%) were of intermediate or low histologic grade and fewer (18%) were high grade. Similarly, most (~97%) of the tumors from both study populations were stage I and II. Small ( < 2 cm) and intermediate (2–5 cm) size tumors were predominant in both studies (98% and 97% for SEARCH and Polish studies, respectively). The majority (70%) of the tumors were invasive ductal carcinomas; however, the Polish study had a substantially higher frequency of ‘other’’ non-ductal or lobular invasive carcinomas than the SEARCH study (28% vs. 7%; *P*-value < 0.001). A higher proportion (61%) of the patients had node-negative than positive (39%) disease, which was slightly fewer in the Polish (56%) than the SEARCH (62%) study population. Only 9% of the patients were HER2 + and this did not differ by study population (*P*-value = 0.12).

**Table 1:** Clinicopathological characteristics of participants in the Polish and SEARCH study populations and overall

					Study population		
	Overall (*N* = 2498)		Polish study (*N* = 558)		SEARCH study (*N* = 1940)		
Characteristic	*N*	%	*N*	%	*N*	%	*P*-value*
Age at diagnosis, years							
<35	41	1.6	3	0.5	38	2.0	<0.001
35–50	804	32.2	148	26.5	656	33.8	
50–65	1342	53.7	272	48.7	1070	55.2	
>65	311	12.4	135	24.2	176	9.1	
Grade							
Low	590	26.1	148	26.5	442	26.0	<0.001
Intermediate	1259	55.7	342	61.3	917	53.8	
High	412	18.2	68	12.2	344	20.2	
Stage							
I	1168	48.9	233	48.8	935	48.9	0.45
II	1154	48.3	231	48.4	923	48.3	
III	50	2.1	12	2.5	38	2.0	
IV	17	0.7	1	0.2	16	0.8	
Morphology							
Ductal	1632	68.6	281	50.3	1351	74.2	<0.001
Lobular	464	19.5	121	21.7	343	18.8	
Other^a^	284	11.9	156	28.0	128	7.0	
Size							
<2 cm	1571	65.5	331	59.3	1240	67.4	0.002
2–5 cm	770	32.1	210	37.6	560	30.4	
>5 cm	58	2.4	17	3.0	41	2.2	
Node status							
Negative	1370	60.9	308	56.0	1062	62.5	0.007
Positive	880	39.1	242	44.0	638	37.5	
Breast cancer subtype							
Luminal A-like	1198	55.7	206	38.0	992	61.7	
Luminal B-like	951	44.3	336	62.0	615	38.3	<0.001
Endocrine therapy							
No	394	19.6	191	35.6	203	13.8	<0.001
Yes	1614	80.4	346	64.4	1268	86.2	

*P-values were from chi-square tests comparing the distributions of the clinicopathological characteristics between the two study populations

^a^In the Polish study, “Other” morphology comprised invasive ductal carcinoma with lobular carcinomatous components (71%), tubular carcinoma (18%), infiltrating papillary carcinoma (7%), and mucinous adenocarcinoma (4%) while in the SEARCH study, the majority (58%) were invasive ductal carcinoma with lobular carcinomatous components, mucinous carcinoma (15%), cribriform carcinoma (13%), adenocarcinoma (not otherwise specified) (9%), and Medullary carcinoma (5%)

### Dynamic range of image analysis-based scores for immunohistochemistry markers in the IHC4-score

Image analysis produced quantitative scores (0–100%) for each of the three nuclear markers (Supplementary Fig. 1) with median (standard deviation) scores of 62% (34), 57% (38), and 9% (11) for ER, PR, and KI67, respectively. When combined with data on HER2 in the IHC4 algorithm, these markers produced an IHC4-score with a dynamic range of −148 to 289 (mean = 33, standard deviation = 65; Supplementary Fig. 2A). The distribution of the IHC4-score differed by study population, with patients from the Polish study generally having higher values than those from the SEARCH study population (Supplementary Fig. 2B).

### Associations between Subtype, IHC4-score, *C*-score, and PREDICT-score with 10-year breast cancer-specific survival

**Fig. 2:**
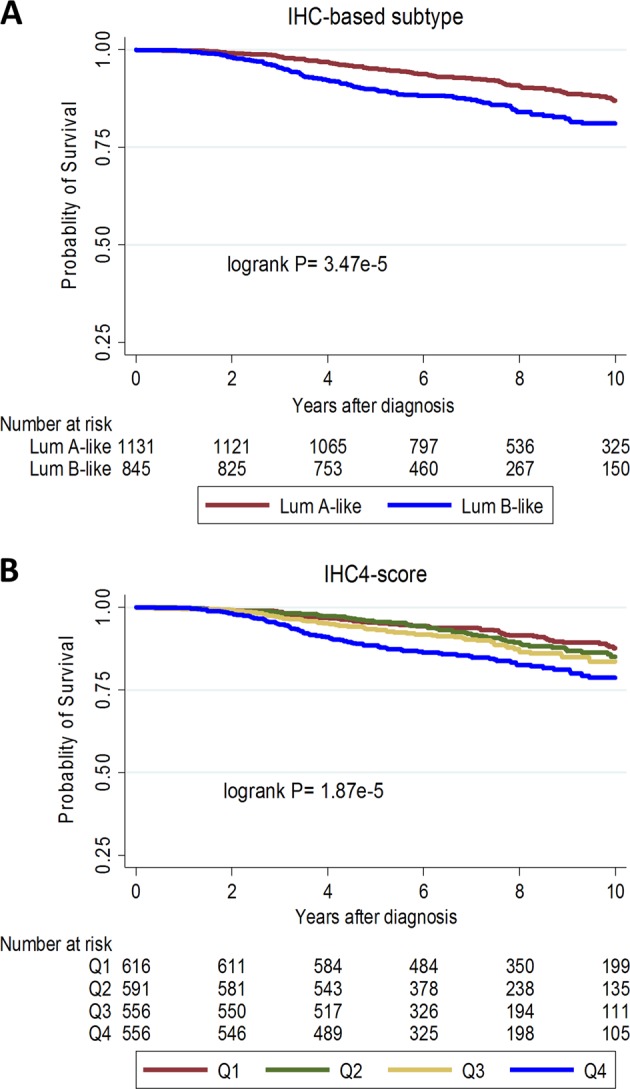
Kaplan–Meier survival curves for the associations between **a** surrogate immunohistochemistry (IHC)-subtypes of luminal (A-like and B-like) breast cancer and **b** quartiles (Q1–Q4) of the IHC4-score with 10-year breast cancer-specific survival overall

In Kaplan–Meier curves (Fig. 2) and in univariable models (Table 2), Subtype (luminal B-like vs. A-like) [hazard ratio (95% confidence interval) = 1.64 (1.25–2.14); *P*-value < 0.001] and IHC4-score [hazard ratio (95% confidence interval)/1 standard deviation = 1.32 (1.20–1.44); *P*-value < 0.001] were significantly associated with survival overall. However, the IHC4-score (LR*χ*^2^ = 40.1) provided more prognostic information than subtype (LR*χ*^2^ = 23.4). A similar pattern of association was seen in both the Polish and SEARCH study populations (Table 2).

**Table 2:** Hazard ratios and 95% confidence intervals for univariable and multivariable associations between IHC4-score and breast cancer subtypes (luminal B-like vs. A-like) with 10-year breast cancer-specific survival among women in the Polish and SEARCH study populations and in both studies combined

	Polish study			SEARCH study			Combined		
Parameter	Hazard ratio	*P*-value	LR *χ*^2^	Hazard ratio	*P*-value	LR *χ*^2^	Hazard ratio	*P*-value	LR *χ*^2^
Univariable analysis									
Subtype (luminal B-like vs. A-like)	1.75 (0.88–3.48)	0.11	2.8	1.65 (1.23–2.21)	0.001	10.9	1.64 (1.25–2.14)	<0.001	23.4
IHC4-score/1 standard deviation	1.54 (1.27–1.81)	<0.001	13.7	1.28 (1.15–1.41)	<0.001	18.2	1.32 (1.20–1.44)	<0.001	40.1
*C*-score /1 standard deviation	1.73 (1.45–2.02)	<0.001	23.9	1.79 (1.67–1.92)	<0.001	137.9	1.78 (1.67–1.90)	<0.001	168.2
PREDICT-score/1 standard deviation	2.21 (1.67–2.93)	<0.001	27.9	2.41 (2.10–2.77)	<0.001	144.7	2.34 (2.07–2.65)	<0.001	178.5
Multivariable analysis^a^									
IHC4-score/1 standard deviation	1.46 (1.18–1.74)	0.001	9.8	1.25 (1.11–1.39)	<0.001	12.0	1.29 (1.16–1.42)	<0.001	19.1
*C*-score/1 standard deviation	1.62 (1.31–1.93)	<0.001	14.5	1.78 (1.65–1.91)	<0.001	129.1	1.75 (1.63–1.87)	<0.001	140.1
Multivariable analysis^b^									
IHC4-score/1 standard deviation	1.46 (1.17–1.74)	0.002	9.2	1.19 (1.05–1.33)	0.007	7.0	1.24 (1.11–1.37)	<0.001	12.5
PREDICT-score/1 standard deviation	1.91 (1.43–2.56)	<0.001	17.1	2.40 (2.09–2.77)	<0.001	135.7	2.26 (1.99–2.56)	<0.001	147.1
Multivariable analysis^c^									
Subtype (luminal B-like vs. A-like)	1.24 (0.58–2.62)	0.58	0.3	1.51 (1.08–2.12)	0.02	5.7	1.38 (1.02–1.88)	0.04	4.3
PREDICT-score/1 standard deviation	1.94 (1.44–2.76)	<0.001	17.6	2.48 (2.13–2.88)	<0.001	125.1	2.30 (2.01–2.63)	<0.001	136.5
Multivariable (luminal A-like tumors only)									
IHC4-score/1 standard deviation	1.29 (0.69–1.90)	0.34	0.9	1.15 (0.93–1.40)	0.17	1.9	1.20 (0.98–1.43)	0.07	3.1
PREDICT-score/1 standard deviation	1.32 (0.57–3.07)	0.51	3.7	2.33 (1.87–2.90)	<0.001	52.1	2.14 (1.74–2.64)	<0.001	46.1
Multivariable (luminal B-like tumors only)									
IHC4-score/1 standard deviation	1.48 (1.14–1.82)	0.006	6.9	1.14 (0.92–1.36)	0.20	1.6	1.22 (1.03–1.41)	0.01	5.3
PREDICT-score/1 standard deviation	2.21 (1.56–3.12)	<0.001	18.5	2.59 (2.06–3.26)	<0.001	65.7	2.36 (1.96–2.84)	<0.001	80.4

^a^Hazard ratio was mutually adjusted for IHC4-score, *C*-score, age, treatment, and, in the combined model, study

^b^Hazard ratio was mutually adjusted for IHC4-score, age, PREDICT-score (which combines information on lymph-nodal involvement, histologic grade, patient age, tumor size, and mode of detection—because we did not have data on mode of detection, this was not computed), treatment and, in the combined model, study

^c^Hazard ratio was mutually adjusted for subtype, age, PREDICT-score, treatment and, in the combined model, study. LR*χ*^2^  =  likelihood ratio chi-square

Both the *C*-score [hazard ratio (95% confidence interval)/1 standard deviation = 1.78 (1.67–1.90); *P*-value = < 0.001], and PREDICT-score [hazard ratio (95% confidence interval)/1 standard deviation = 2.34 (2.07–2.65); *P*-value < 0.001] were associated with 10-year breast cancer-specific survival, with the model based on PREDICT-score (LR*χ*^2^ = 178.5) fitting better than that based on the *C*-score (LR*χ*^2^ = 168.2). In addition, PREDICT-score provided more prognostic information [∆LR*χ*^2^ = 9.0; *P*-value = 0.002] than the *C*-score [∆LR*χ*^2^ = 5.6; *P*-value = 0.02] in this population, hence it was used as the adjustment factor in multivariable models.

**Fig. 3:**
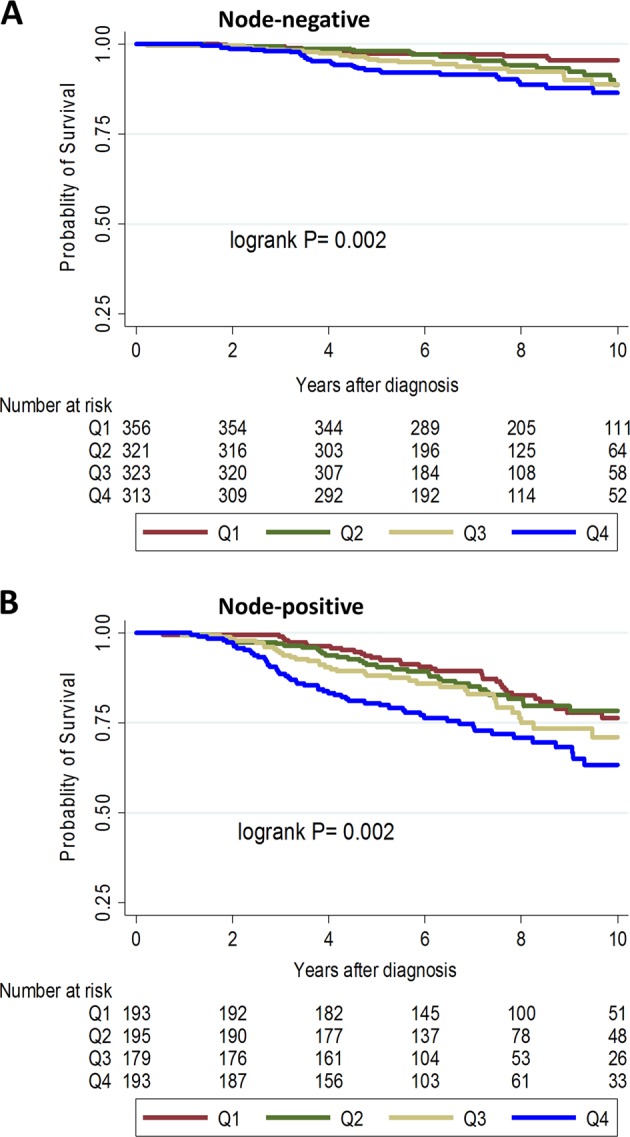
Kaplan–Meier survival curves for the associations between quartiles (Q1–Q4) of the IHC4-score and 10-year breast cancer-specific survival in node-negative (**a**) and node-positive (**b**) luminal-like breast cancer patients

The IHC4-score remained significantly associated with survival, overall [hazard ratio (95% confidence interval)/1 standard deviation = 1.24 (1.11–1.37); *P*-value< 0.001] and in both the Polish [hazard ratio (95% confidence interval)/1 standard deviation = 1.46 (1.17–1.74); *P*-value 0.002] and SEARCH [hazard ratio (95% confidence interval)/SD = 1.19 (1.05–1.33); *P*-value = 0.007] study populations after adjusting for PREDICT-score (Table 2). Further, when we performed analyses stratified by lymph-nodal involvement, higher values of the IHC4-score were associated with worse breast cancer-specific survival in women with node-negative (Fig. 3a; logrank *P*-value = 0.002) and node-positive (Fig. 3b; logrank *P*-value = 0.002) disease.

### Association between IHC4-score and 10-year breast cancer-specific survival within luminal A-like and B-like subtypes of breast cancer

Overall, the IHC4-score was associated with survival in both luminal A-like [hazard ratio (95% confidence interval)/1 standard deviation = 1.20 (0.98–1.43); *P*-value = 0.07) and luminal B-like [hazard ratio (95% confidence interval)/1 standard deviation = 1.22 (1.03–1.41); *P*-value = 0.01] subtypes (*P*-value for heterogeneity = 0.97). Although the hazard ratio estimate was slightly attenuated in luminal A-like [hazard ratio (95% confidence interval)/1 standard deviation = 1.10 (0.88, 1.33); *P*-value = 0.37)] subtype defined using 1% threshold for ER and PR, the estimates remained essentially the same for luminal B-like tumors [hazard ratio (95% confidence interval)/1 standard deviation = 1.22 (1.03–1.41); *P*-value = 0.02].

**Fig. 4:**
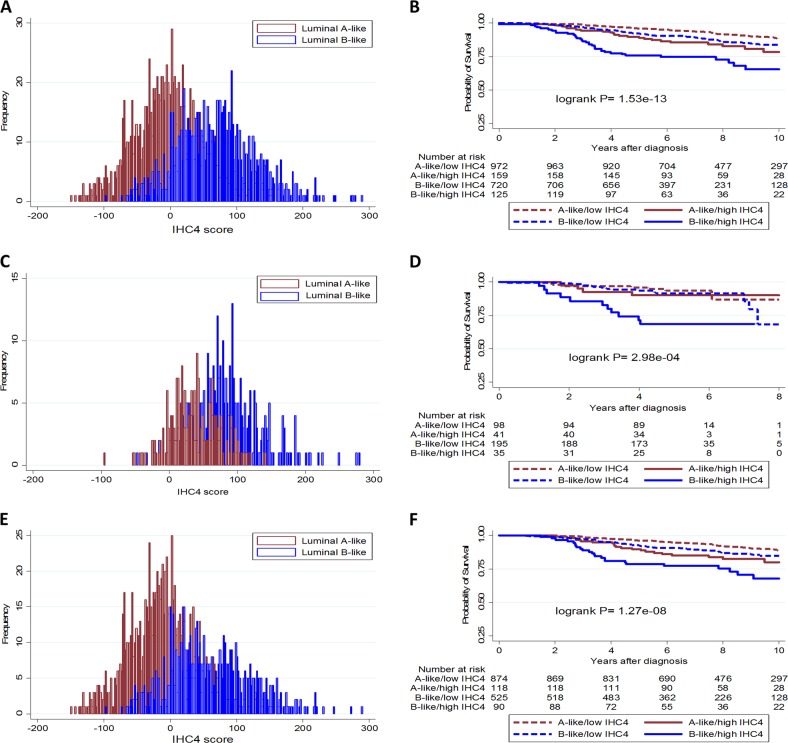
Distribution of the IHC4-score in luminal A-like and B-like subtypes of breast cancer and Kaplan–Meier survival curves for the associations between subtypes of luminal-like breast cancer stratified by levels of IHC4-score, overall (**a** and **b**), and among patients in the Polish (**c** and **d**) and SEARCH (**e** and **f**) study populations

We observed an overlap in the distribution of the IHC4-score between luminal A-like and B-like subtypes, overall and in both study populations (Fig. 4). Thus, by comparing women with IHC4-score above the mean + 1 standard deviation (denoted as high IHC4-score) with those that had scores below this threshold (low IHC4-score), we observed those with high IHC4-score to have significantly worse survival outcomes than those with low scores, overall and in both study populations (Fig. 4). Following adjustment for the PREDICT-score in Cox proportional hazard models, we observed differences in high vs. low IHC-score categories in the luminal B-like subtype overall and in both the Polish and SEARCH study populations (Table 3). However, differences did not attain statistical significance in the luminal A-like subtype in the Polish study, which is likely due to the limited number of events (number (deaths/cases) = 7/99 and 5/41 in the low and high IHC4-score categories, respectively) in this sub-population (Table 3).

**Table 3:** Hazard ratios and 95% confidence intervals for the associations between categories of the IHC4-score and 10-year breast cancer-specific survival among women with luminal A-like and B-like breast cancer, overall and by study population

Subtype/IHC4-score	Cases/events	Hazard ratio^a^	*P*-value
Overall			
Luminal A-like			
Low IHC4-score	973/100	1.00 (reference)	
High IHC4-score	159/27	2.23 (1.36–3.65)	0.001
Luminal B-like			
Low IHC4-score	720/90	1.00 (reference)	
High IHC4-score	125/36	2.41 (1.57–3.68)	<0.001
B-like/low IHC4-score	720/90	1.00 (reference)	
A-like/high IHC4-score	159/27	1.64 (1.02–2.65)	0.04
Polish study			
Luminal A-like			
Low IHC4-score	99/7	1.00 (reference)	
High IHC4-score	41/5	1.81 (0.50, 6.48)	0.36
Luminal B-like			
Low IHC4-score	195/20	1.00 (reference)	
High IHC4-score	35/11	3.63 (1.71, 7.73)	0.001
SEARCH study			
Luminal A-like			
Low IHC4-score	874/93	1.00 (reference)	
High IHC4-score	118/22	2.16 (1.25, 3.75)	0.006
Luminal B-like			
Low IHC4-score	525/70	1.00 (reference)	
High IHC4-score	90/25	2.03 (1.21, 3.43)	0.008

^a^Hazard ratio was mutually adjusted for age, PREDICT-score (which combines information on lymph-nodal involvement, histologic grade, patient age, tumor size, and mode of detection— because we did not have data on mode of detection, this was not computed), treatment, and study population. Categories (high and low) of the IHC4-score were obtained by dichotomization of the data at the mean + 1 standard deviation threshold.

## Discussion

In the current analysis, we combined quantitative scores of ER, PR, HER2, and KI67 in the IHC4-score and compared its prognostic performance with categorical combinations defining A-like and B-like subtypes of luminal-like breast cancer. We also investigated the prognostic value of the IHC4-score in relation to other clinical prognostic factors, combined in the *C*-score and PREDICT-score. Our findings show that the IHC4-score provided more prognostic information than immunohistochemistry-based subtyping of luminal-like breast cancer. Additionally, the IHC4-score was associated with survival in both the luminal A-like and B-like subtypes after adjusting for the PREDICT-score, which provided more prognostic information than the *C*-score in this study population. Our findings also suggest that the dynamic range of the IHC4-score can be leveraged to provide prognostic information in both node-negative and node-positive disease and to further stratify women with luminal A-like or B-like breast cancer into subgroups with different prognoses.

Successive St. Gallen panels [18, 20, 29] have endorsed the use of immunohistochemical markers for the surrogate definition of the A-like and B-like subtypes of luminal-like breast cancer for deciding systemic therapy options. Based on current guidelines [20], most patients with the luminal B-like subtype receive chemotherapy in addition to standard endocrine treatment. Conversely, endocrine therapy is the mainstay of treatment for luminal A-like disease, except for a subset of patients for whom the addition of chemotherapy may be warranted. Some indications for cytotoxic therapy in luminal A-like patients include high 21-gene recurrence score and high-risk status on the 70-gene panel [18, 20]. However, dichotomization of ER, PR, HER2, and KI67 for subtype definition may be associated with the loss of prognostically relevant information. Moreover, intratumor heterogeneity may lead to discordant classifications of breast cancer subtypes since conventional subtyping approaches assign patients into discrete categories based on the topographical region of the tumor that has been sampled [30]. By combining quantitative data on all four markers, the IHC4-score has a dynamic range that can allow for the evaluation of dose–response relationships with survival thereby avoiding the pitfalls associated with assigning patients into discrete categories [13, 31].

We have previously demonstrated the prognostic value of automated scores on the individual immunohistochemical markers that are currently used to define breast cancer subtypes [23, 25]. However, the prognostic significance of combining automated scores on all four markers has not been previously studied. In this analysis, we showed that a combined score of these markers is significantly associated with 10-year breast cancer-specific survival even after adjustment for the PREDICT-score. This finding is particularly relevant given that, unlike expression-based assays, the IHC4-score and PREDICT-score are based on routinely determined clinicopathological and immunohistochemical parameters in clinical practice thereby making them potentially available to many patients with luminal-like breast cancer. Although multiparameter molecular tests [14–17] also quantify the expression of several genes, including those related to ER, PR, HER2, and proliferation, these are expensive, not widely available, and it remains unclear whether they provide additional prognostic information to PREDICT.

Overall, the range of clinical applications of the IHC4-score is still evolving [32–34]. One previous study documented its capacity to distinguish breast cancer patients with intermediate Nottingham Prognostic Index into subgroups with low and high-risk of recurrence [32, 33]. Our findings from the current study provide clues into other potential uses of the IHC4-score in clinical practice. For instance, the finding of significant associations with survival for the IHC4-score and PREDICT-score in luminal A-like and B-like patients suggest that chemotherapy recommendations should be based on its predicted absolute benefit regardless of immunohistochemistry-based subtype. In addition, the overlap in the distribution of the IHC4-score between A-like and B-like tumors that we observed may be indicative of the need to leverage quantitative information on immunohistochemical markers to provide additional prognostic information beyond what is contained in immunohistochemistry-based subtypes.

Despite its potential benefits, the widespread adoption of the IHC4-score may be affected by concerns regarding its analytical validity. There is the perception that immunohistochemical methods lack reproducibility and suffer from variable degrees of between-laboratory discordance. However, Dodson et al. [35] showed in a recent multi-institutional analytical validity study that risk of recurrence estimates with the IHC4 + *C*-score were tolerant of variations in staining and scoring across different laboratories. Moreover, several international efforts have led to the publication of guidelines that will help to enhance the validity of assays performed in laboratories across the globe [36–38].

An important strength of this study was that we used a digital image analysis-based approach for the centralized scoring of all four immunohistochemical markers, which yielded quantitative scores on ER, PR, and KI67. Although visual scoring by a trained expert can guarantee accurate discrimination between epithelial and stromal cells and between malignant and benign epithelial cells, this method is labor intensive and suffers from varying degrees of intra- and inter-observer discordance [21, 22]. In contrast, image analysis-based methods are high-throughput, highly reproducible, and show good agreement with pathologist’s-based scores [23, 24, 39–44]. Previous studies looking at the IHC4-score have focused on its prognostic performance in luminal-like breast cancer as a homogeneous entity and none has evaluated its prognostic value in relation to PREDICT-score. To the best of our knowledge, ours is the first study to specifically investigate the prognostic value of the IHC4-score in relation to subtypes of luminal-like breast cancer, as well as the PREDICT-score. Furthermore, our analysis involved patients from two study populations for whom the IHC4-score had never been applied, which allowed us to compare the results across populations.

In terms of limitations, despite their promise as alternatives to visual scoring, concerns exist regarding the accuracy of image analysis-based methods in discriminating between malignant and benign epithelial cells. We have previously documented the underestimation of hazard ratio estimates when comparing image analysis with pathologist’s-based scores [23, 24]. This was due to the attenuation of the performance of the image analysis algorithm in the presence of mixed cell populations. However, we utilized tissue microarrays for this study, which may limit the impact of mixed cell populations on our results since cores on tissue microarrays are typically enriched for tumor cells. Also, at the time most of our patients were recruited, ER + and PR + tumors were defined based on Allred score of > 2, corresponding to a proportion score of > 10%. However, this threshold has evolved over time, with current recommendations stipulating a cutoff point of ≥ 1% [20]. Nonetheless, many studies still utilize the 10% threshold. Moreover, when we redefined subtypes based on the 1% threshold as part of sensitivity analysis our results remained essentially the same.

This study was not designed to assess the predictive value of the IHC4-score for chemotherapy response. In view of results from a few studies showing poor chemotherapy response in high-risk luminal A-like tumors [45], an important area of future research will be the determination of the predictive value of the IHC4-score for chemotherapy response in patients with luminal A-like disease. Interestingly, recent findings suggest that the Magee Equation [46], another inexpensive tool that is based on ER, PR, HER2, and KI67 in addition to the Nottingham score and tumor size, can be used to predict pathologic response to neoadjuvant chemotherapy in ER + /HER2-negative/equivocal breast cancer [47]. The PREDICT-score also provides information on estimated treatment benefit for both ER + and ER- breast cancer patients. However, both the Magee Equation and PREDICT-score are based on visual assessments of all four immunohistochemical markers and it remains unclear whether the incorporation of automated measures can help refine the discriminatory accuracy of both tools, particularly in women with equivocal scores.

In conclusion, findings from this study showed that quantitative measures of ER, PR, HER2, and KI67, combined in the IHC4-score, provided more prognostic information than categorical combinations in immunohistochemistry-based subtypes of luminal-like breast cancer. In addition, the IHC4-score was associated with 10-year breast cancer-specific survival in patients with both luminal A-like and B-like tumors even after accounting for PREDICT-score, which was the strongest prognostic factor in this population. Taken together, these findings support the view that the IHC4-score can be used as an inexpensive adjunct to other clinical prognostication tools to aid treatment decision-making in patients with luminal-like breast cancer, irrespective of subtype. Given the prognostic strength of the PREDICT-score that we observed, further studies will be needed to determine whether combining the IHC4-score and PREDICT-score will provide superior prognostic information than PREDICT-score plus HER2 and KI67.

## Supplementary information

Supplementary Table 1

Supplementary Figure 1

Supplementary Figure 2

OPEN ACCESS APC FORM
