# Supplementary material for: Combined quantitative measures of ER, PR, HER2, and KI67 provide more prognostic information than categorical combinations in luminal breast cancer
Source: Mod Pathol. 2019 Apr 11;32(9):1244–56. doi: 10.1038/s41379-019-0270-4 (PMC6731159; doi:10.1038/s41379-019-0270-4)
Supplement: Supplementary file 1 — Supplementary Table 1 [file 41379_2019_270_MOESM1_ESM.docx]

**Supplementary Materials**

**Supplementary Table 1:** Immunohistochemical procedures for ER, PR, HER2 and KI67 by study

**Supplementary Figure 1:** Distribution of image analysis-based scores for ER (A), PR (B), KI67 (C); and HER2 (D).

**Supplementary Figure 2:** Distribution of quantitative image-analysis based IHC4-score, overall (A) and by study population (B).
