# Supplementary material for: Combined quantitative measures of ER, PR, HER2, and KI67 provide more prognostic information than categorical combinations in luminal breast cancer
Source: Mod Pathol. 2019 Apr 11;32(9):1244–56. doi: 10.1038/s41379-019-0270-4 (PMC6731159; doi:10.1038/s41379-019-0270-4)
Supplement: Supplementary file 2 — Supplementary Figure 1 [file 41379_2019_270_MOESM2_ESM.docx]

**Supplementary Table 1**: Immunohistochemical procedures for ER, PR, HER2 and KI67 by study

| **Marker** | **Study** | **Processing Laboratory** | **Clone** | **Clonality** | **Supplier** | **Dilution** | **Antigen retrieval** |
| --- | --- | --- | --- | --- | --- | --- | --- |
| ER | SEARCH | Cancer Research Institute Cambridge, UK | 6F11/2 | Mouse monoclonal | Novocastra | 1 in 70 | Citrate buffer pH6, 30 min |
| ER | PBCS | National Cancer Institute, USA | 6F11/2 | Mouse monoclonal | Novocastra | 1 in 200 |  |
| PR | SEARCH | Cancer Research Institute Cambridge, UK | PR 636 | Mouse monoclonal | Dako | 1 in 50 | Citrate buffer pH6, 30 min |
| PR | PBCS | National Cancer Institute, USA | PR 636 | Mouse monoclonal | Dako | 1 in 1000 |  |
| KI67 | SEARCH | Cancer Research Institute Cambridge, UK | MIB-1 | Mouse monoclonal | Dako | 1:200 | Tris-EDTA buffer, ph9, 30 min |
| KI67 | PBCS | National Cancer Institute, USA | MIB-1 | Mouse monoclonal | Dako | 1:500 | Tris-EDTA buffer, Ph9, 20 min |
| HER2 | SEARCH | Addenbrooks Hospital, Cambridge, UK | Herceptest kit K5207 | Humanized monoclonal | Dako |  | Citrate buffer pH6, 40 min |
| HER2 | PBCS | National Cancer Institute, USA | Herceptest kit K5207 | Humanized monoclonal | Dako | 1 in 2000 |  |
