# Supplementary figures and images for: Combined quantitative measures of ER, PR, HER2, and KI67 provide more prognostic information than categorical combinations in luminal breast cancer
Source: Mod Pathol. 2019 Apr 11;32(9):1244–56. doi: 10.1038/s41379-019-0270-4 (PMC6731159; doi:10.1038/s41379-019-0270-4)

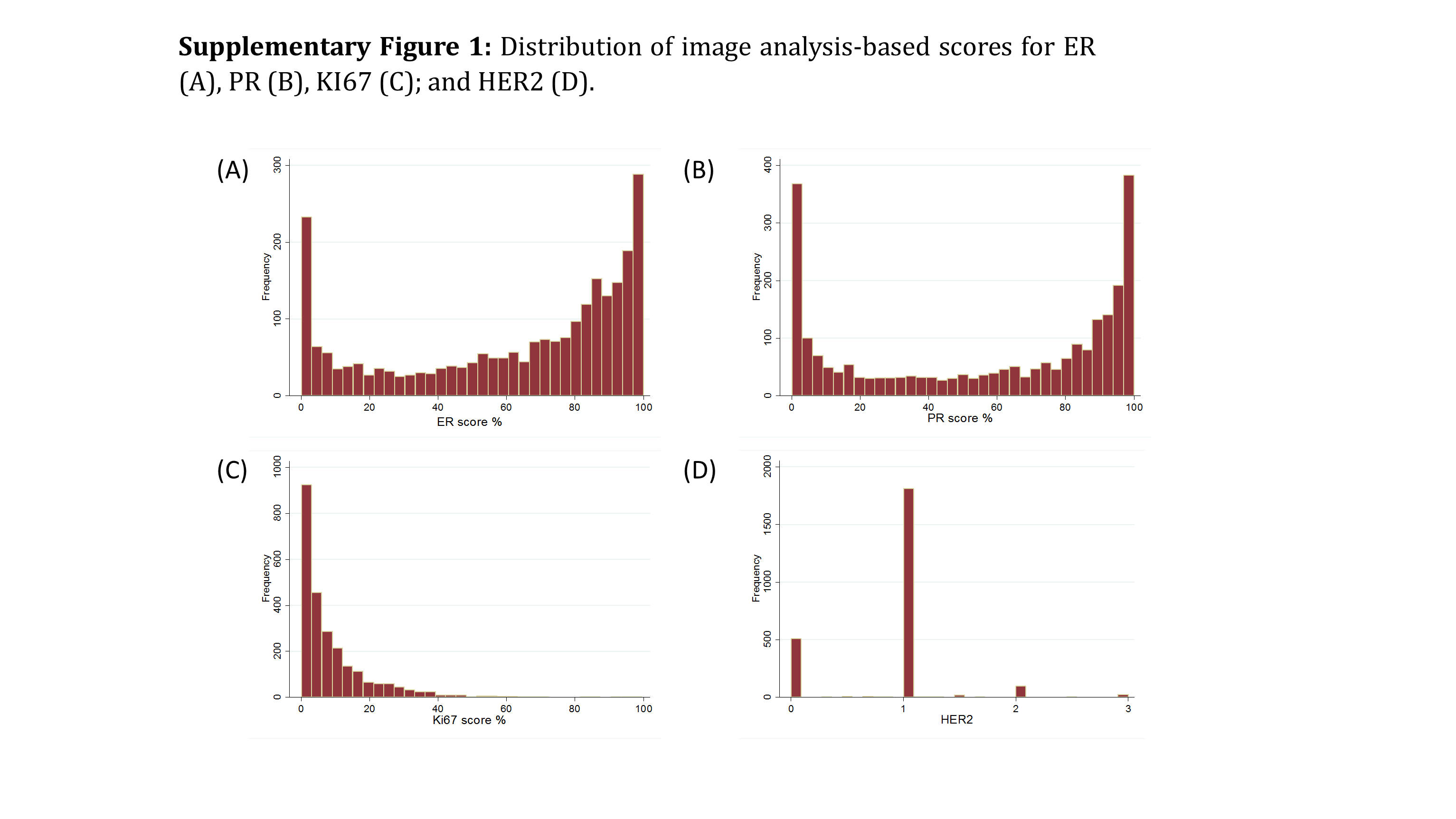

Supplement: Supplementary file 3 — Supplementary Figure 2 [file 41379_2019_270_MOESM3_ESM.tif]

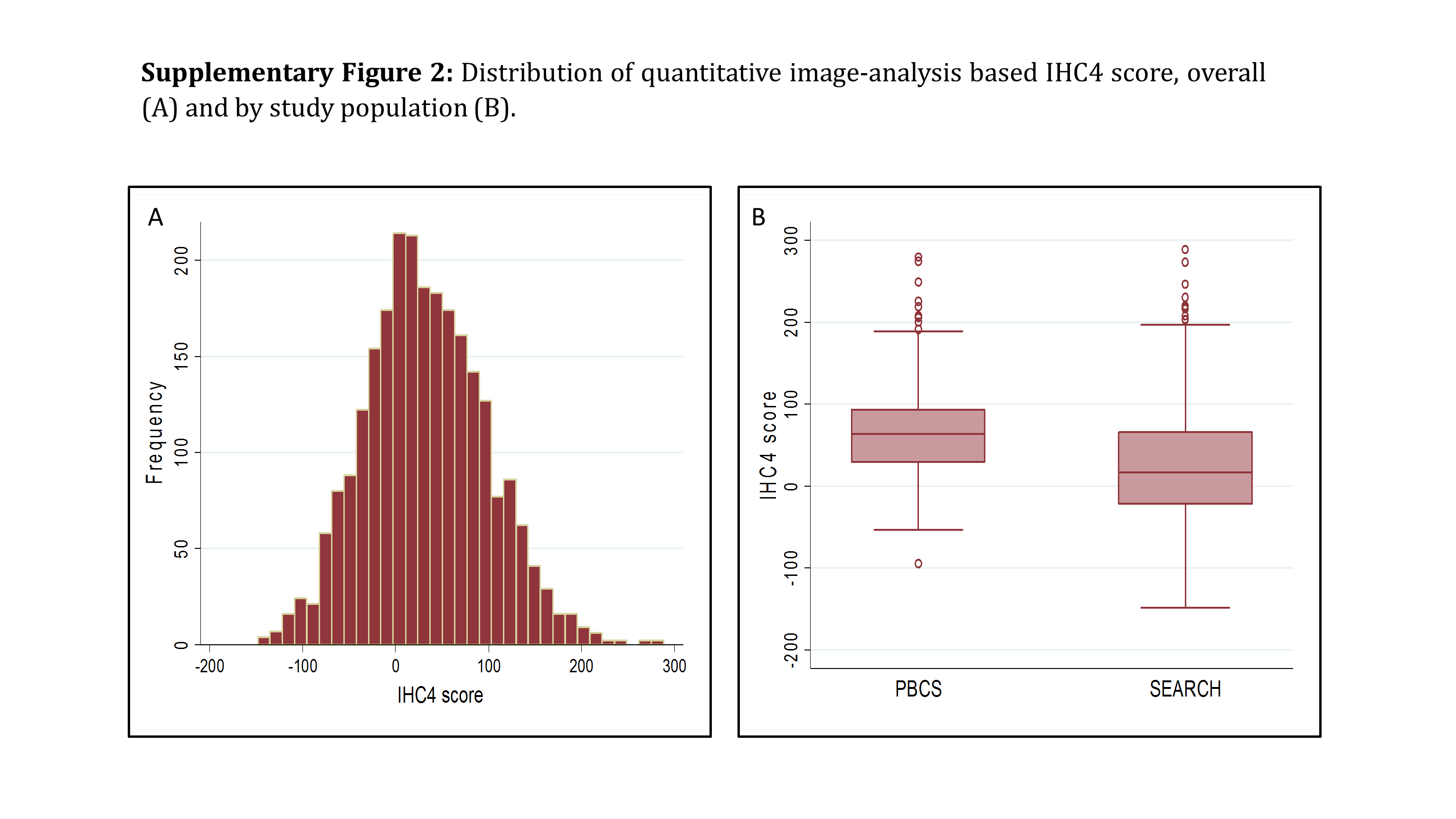

Supplement: Supplementary file 4 — OPEN ACCESS APC FORM [file 41379_2019_270_MOESM4_ESM.tif]
